# Minireview: Novel Micropeptide Discovery by Proteomics and Deep Sequencing Methods

**DOI:** 10.3389/fgene.2021.651485

**Published:** 2021-05-06

**Authors:** Ravi Tharakan, Akira Sawa

**Affiliations:** ^1^National Institute on Aging, National Institutes of Health, Baltimore, MD, United States; ^2^Departments of Psychiatry, Neuroscience, Biomedical Engineering, and Genetic Medicine, Johns Hopkins University School of Medicine, Baltimore, MD, United States; ^3^Department of Mental Health, Johns Hopkins Bloomberg School of Public Health, Baltimore, MD, United States

**Keywords:** micropeptides, miniproteins, proteogenomics, sORF, ribosome profiling, proteomics, genomics, RNA sequencing

## Abstract

A novel class of small proteins, called micropeptides, has recently been discovered in the genome. These proteins, which have been found to play important roles in many physiological and cellular systems, are shorter than 100 amino acids and were overlooked during previous genome annotations. Discovery and characterization of more micropeptides has been ongoing, often using -omics methods such as proteomics, RNA sequencing, and ribosome profiling. In this review, we survey the recent advances in the micropeptides field and describe the methodological and conceptual challenges facing future micropeptide endeavors.

## Introduction

The sequencing and publication of complete genomic sequences of many organisms have aided the medical sciences greatly, allowing advances in both human genetics and the biology of human disease, as well as a greater understanding of the biology of human pathogens (Firth and Lipkin, 2013). In particular, the human genome sequence has advanced human disease genetics by allowing genome-wide association studies (Hofker et al., 2014) (GWAS), and knowledge of gene sequences and their chromosomal loci has produced a deeper understanding of the biology of all organisms whose genomes have been sequenced, including human pathogens such as viruses (Lu et al., 2020). Crucial to all of these efforts is genome annotation, which uses genomic, genetic, epigenetic, and other information to find loci in the genome which code functional genes (Salzberg, 2019). While genome annotation has been performed alongside efforts to sequence genomes, the continuing pace of novel gene discovery, aided by advancing technology in molecular biology and biochemistry, suggests that annotation is often incomplete.

During the early stages of genome annotation, when genomes such as the human genome were first sequenced, a lower limit was placed on the length of an open reading frame (ORF) that could be considered a possible gene (Dujon et al., 1994). These limits were set by modeling a biochemically equivalent random genome and determining the ORF length distribution over that random genome, thus producing a length distribution of “random” ORFs. In the case of the human genome, in order to exclude such random ORFs, the minimum ORF length was set to be 100 codons by reasoning about the size distribution of random ORFs (Dujon et al., 1994). However, while a high proportion of ORFs below these limits may be spurious, there are also a substantial number which are real genes, and these are missed by such a filtering step (Basrai et al., 1997). Furthermore, while gene discovery can be accomplished by means other than genome annotation, it appears that these original annotation decisions continue to be reflected in our current understanding of genomes, since some human protein databases contain a disproportionately low number of genes annotated below the 100 codon cutoff (Frith et al., 2006).

Recently, evidence has emerged that many of these short (<100 codons) open reading frames may indeed be protein-coding genes, whose gene products have been named “micropeptides” (Couso, 2015). Evidence for functionality of these short peptides has come from several sources, including bioinformatics, through novel conservation analyses (Crowe et al., 2006), and biochemical approaches, such as expressed sequence tag experiments (Frith et al., 2006), deep sequencing based experiments such as RNA sequencing (Kageyama et al., 2011) and ribosome profiling (Ingolia et al., 2011), as well as proteomics (Slavoff et al., 2013, 2014; Khitun and Slavoff, 2019; Cao et al., 2020). These micropeptides have been found in studies of many model organisms, suggesting that micropeptide genes indeed exist throughout all genomes. Nevertheless, it has been controversial how many micropeptide genes there are, and general estimates have varied by orders of magnitude (Andrews and Rothnagel, 2014). In particular, since all techniques have biases and false positives, there has been continued debate on the extent to which evidence for micropeptides is artifact of the techniques used for discovery (Guttman et al., 2013; Ingolia et al., 2014). Furthermore, the mechanisms by which micropeptides perform their functions in the cell is often difficult to determine; in particular, it is still not clear whether micropeptides, as a class, share a general cellular role, or whether they have diverse functions in the same way large well-annotated proteins do (Couso and Patraquim, 2017). In this review, we will examine some of the recent developments in the field of micropeptide discovery, with a special emphasis on mass spectrometry-based approaches to the study of micropeptides.

## Micropeptide Discovery in Lower-Order Organisms

In prokaryotes, annotation of sORF-encoded micropeptides has been of increasing interest. In the bacterium *Mycoplasma pneumoniae*, micropeptides translated from ncRNA were discovered by mass spectrometry, defining the total complement of micropeptides in that organism’s genome at 67, approximately 5% of all coding genes (Lluch-Senar et al., 2015). A transposon-based essentiality screen found that 53% of these micropeptides were essential for the bacterium’s growth, indicating that many micropeptides are not only functional but essential for bacterial growth. Since *M. pneumoniae* has a relatively small genome of 816 kb which is likely to be well annotated, such an essentiality study also suggests that many micropeptides are proteins essential for a minimal organism and thus essential for life (Lluch-Senar et al., 2015). Finally, in the widely used model bacterium *Escherichia coli*, one study discovered 44 micropeptides, many of which also perform basic functions in the cell (Hemm et al., 2008), while a more recent study found 36, using an epitope tagging method (VanOrsdel et al., 2018).

Micropeptide discovery has also continued apace in eukaryotic model systems. A study in *Saccharomyces cerevisiae* by ribosome profiling found many cases of translation of micropeptides, which appear to serve some purpose during the meiotic process (Brar et al., 2012). During meiosis, translation of some 9,989 unannotated ORFs was found, and these novel genes appeared to have their translation increased in a regulated fashion during meiosis, indicating once again that they are functional, although the functions of these many putative novel genes have not been defined yet (Brar et al., 2012). A bioinformatics screen of the yeast genome searching for micropeptides similarly yielded 184 yeast sORFs conserved across many species, suggesting that they may be functional (Kastenmayer et al., 2006). These novel genes were then validated as to function by development of deletion strains for 140 of them, of which nine gave clearly observable phenotypes. The latter study provides a model of how novel micropeptide genes can be validated as to function; furthermore, the large increase in the number of micropeptides observed between the two studies demonstrates the role of advancing technology in the rapid advance of micropeptide annotation.

## Human-Relevant Micropeptide Discovery in Model Systems

Studies in higher-order animals have also found several significant novel micropeptides. Very relevantly for human health, a screen in *Danio rerio* discovered a novel micropeptide translated from a transcript annotated as a long non-coding transcript (lncRNA), naming the micropeptide *Toddler* (Pauli et al., 2014). This micropeptide was discovered by a series of screens, in which developing zebrafish were subjected to RNA sequencing to discover novel lncRNAs (Chew et al., 2013), and the same material was used for a ribosome profiling study (Pauli et al., 2012). The latter ribosome profiling study showed that 399 of the putative lncRNAs were indeed translated, of which one produced a micropeptide confirmed by mass spectrometry. GFP tagging of the novel micropeptide to find tissue distribution and a knockout animal model showed that the micropeptide appears to function as an extracellular secreted ligand for the Apelin receptor, essential for cell migration during embryonic development (Pauli et al., 2014). Importantly, the same micropeptide was found to be essential for regulation of the human cardiovascular system, again functioning as a ligand for the Apelin receptor (Yang et al., 2017). This micropeptide has been found to be involved in pre-eclampsia pathology in a mouse model (Ho et al., 2017). As GPCRs are very frequently targeted by drugs, the discovery of this novel ligand could eventually produce novel therapies, as has been recently proposed (Kuba et al., 2019). Indeed, while no other micropeptides have so far been found to be GPCR ligands, the *Toddler* peptide shows that it may be productive to screen sets of detected micropeptides for GPCR activity. For example, hits from mass spectrometry searches could be tested for activity against GPCRs, as has been done for peptide libraries before (Zhang et al., 2015; Yaginuma et al., 2019). Furthermore, this series of studies thus shows that discoveries of micropeptides in animals cannot only be relevant for human biology, they can also almost immediately lead to novel therapies.

In another animal model system, *Drosophila melanogaster*, progress in annotation of micropeptides has been even more rapid. Several screens of this system have produced a number of novel micropeptides which regulate the cardiovascular system (Magny et al., 2013), developmental regulation through proteasomal function (Zanet et al., 2015), and control of RNA polymerase (Hanyu-Nakamura et al., 2008). In the cardiovascular system, the micropeptides Sarcolamban A and B were initially found in a search for functional short ORFs (sORFs) among the set of putative ncRNAs, a screen which found two possible micropeptides of 28 and 29 amino acids long on a single transcript (Magny et al., 2013). *In vivo* translation and GFP tagging confirmed translation localized to the sarcoplasmic reticulum, and a null mutant showed a cardiac arrhythmia phenotype with dysregulated calcium transients, suggesting a novel micropeptide involved in regulation of the SERCA pump. Strikingly, the micropeptides were found to be highly conserved throughout evolution, including in humans (Magny et al., 2013). In *Drosophila* development, the *mlpt/tal/pri* gene, discovered independently by several groups, contains four sORFs which appear to code for peptides (Zanet et al., 2016). Null mutants of this gene show dramatically dysregulated development, apparently due to disruption of a transcription factor (Zanet et al., 2015). In this case, the micropeptides appear in some fashion to regulate the ubiquitination and proteasomal degradation of the transcription factor. These examples, unlike the above *Toddler* example, show cases of intracellular micropeptides regulating protein-protein interactions. Finally, a genome-wide study by ribosome profiling has increased the number of candidate micropeptides in the *Drosophila* genome to ∼285, although most of these are of unknown function (Aspden et al., 2014; Zanet et al., 2016).

In mouse and human, finally, there has also been progress in identifying biologically relevant micropeptides, including the case of the *Toddler* peptide described above. Next discovered was the *Myoregulin* micropeptide, which followed on from the discovery of *Sarcolamban* in *Drosophila* (Anderson et al., 2015). Like *Sarcolamban*, the *Myoregulin* micropeptide regulates activity of the SERCA pump and thereby calcium transients, and similarly, it was found by bioinformatically screening newly discovered non-coding RNA transcripts for short ORFs which could encode putative micropeptides. The peptide was found by labeling experiments to interact with the SERCA pump and have some homology to *Sarcolamban*, as well as to the human peptides *Phospholamban* and *Sarcolipin* (Anderson et al., 2015). Further extending work on the *Myoregulin* micropeptide, three more micropeptide members of the same family were found by screening the peptide-binding motif of the family, discovering the micropeptides *Dworf* (Nelson et al., 2016), *Endoregulin*, and *Another-regulin* (Anderson et al., 2016). These peptides all regulate SERCA, and their tissue distribution is substantially different, including tissues beyond muscle (Anderson et al., 2016), indicating that micropeptide-SERCA interactions are a widespread and perhaps fundamental system for regulating calcium transients in mouse and human.

Besides bioinformatics screens of ORFs in ncRNAs, the human and mouse systems have also been substantially probed for micropeptides using ribosome profiling and mass spectrometry. A ribosome profiling study in mouse embryonic stem cells found that many short ORFs are translated in these cells, and the majority of lncRNAs have translation levels comparable with annotated protein-coding genes, suggesting that many lncRNAs in fact encode micropeptides (Ingolia et al., 2011). The same study also observed widespread translation in upstream ORFs (uORFs) of known coding genes, which translation was downregulated during embryoid body formation. However, these latter observations cannot necessarily be taken to indicate functional micropeptides, since ribosome presence on a transcript means only that the ribosome is bound, but not that a functional peptide is produced. For example, ribosome presence on upstream ORFs has canonically been interpreted as a regulatory process by which the ribosome’s access to the main coding ORF is blocked, thus impeding translation of the main ORF (Hinnebusch, 2014). These uORF peptides, however, have been recently found to be presented on MHC molecules, suggesting a potential function in human immunity (Starck et al., 2016). Mass spectrometry-based discovery of novel micropeptides has been successfully performed by peptidomics studies on human cell lines. In this approach, small peptides are purified by size exclusion from the larger proteome, and these small peptides are then analyzed by mass spectrometry (Slavoff et al., 2013). This approach was successfully applied to find 90 novel micropeptides, including NoBody (D’Lima et al., 2017), a small peptide that appears to downregulate mRNA processing granule formation and participate in RNA decapping; and MRI-2, which appears to participate in DNA repair (Slavoff et al., 2014).

Finally, a very large-scale search for micropeptides in human cells used a combination of ribosome profiling and mass spectrometry to generate potentially translated micropeptides and CRISPR knockouts to validate the micropeptides as functional (Chen et al., 2020), resulting in some 570 novel micropeptides. In this study, ribosome profiling was performed on several cell lines in order to find novel coding regions, resulting in 3,455 novel coding regions, as well as 2,466 extensions of known coding regions. Very few of the peptides encoded by these novel regions were detected by mass spectrometry, but the authors then developed a CRISPR-based screen to validate the peptides functionally, constructing an sgRNA library targeting 2,353 of the putative novel regions, and testing for the effect of the CRISPR knockout on cell growth. Several hundred of the micropeptide knockouts showed phenotypes, suggesting functional micropeptides. CRISPR knockouts disrupt the DNA, and so it is not possible to distinguish between a functional non-coding RNA and a functional micropeptide, because both the RNA and protein level will be disrupted. A solution for this is to use CRISPR to mutate only the start codon of the micropeptide, which will allow a non-coding RNA to function but block expression of the micropeptide (Chen et al., 2020).

Since ribosome profiling and mass spectrometry have so far been the most successful methods by which mammalian micropeptides have been discovered, we have recently proposed combining the two methods (Tharakan et al., 2020), in a so-called proteogenomics approach (Nesvizhskii, 2014). In these studies, a combination of RNA sequencing and proteomics data are used, where the RNA sequencing database is used to assemble a transcriptome, which is then translated in six frames to yield a database of all possible proteins and peptides. Several groups have attempted this approach in human samples, most notably in the TCGA project (Cancer Genome Atlas Network, 2012; Wang and Zhang, 2013) and other projects (Wang et al., 2019). The central problem with these databases is their extremely large size, usually on the order of several million candidate proteins. Databases with a very large size will decrease the sensitivity of the search. To solve this problem, we propose using ribosome profiling data to filter the candidate protein list before the final analysis. This proteogenomics approach, of combining RNA sequencing, ribosome profiling, and mass spectrometry (Tharakan et al., 2020), can also be used to identify so-called tumor “neoantigens,” which are mutated proteins produced by tumors (Schumacher and Schreiber, 2015). Once again, exome sequencing or RNAseq of tumors produces databases that are too large, which can be reduced by filtering through ribosome profiling.

Several micropeptides involved in the regulation of human metabolism have been found. Using mass spectrometry, the micropeptide SPAR, small regulatory polypeptide of amino acid response, was discovered by a proteomic analysis of a human cell line (Matsumoto et al., 2017), and found to regulate mTOR function. Similarly, the micropeptides mitoregulin (Stein et al., 2018) and MOXI (Makarewich et al., 2018) which both regulate mitochondrial function, were found through bioinformatics methods coupled with experimental validation. Functional micropeptides have also been found in the human mitochondrial genome. The *Humanin* peptide, encoded by a short ORF from the mitochondrial DNA, was discovered years ago by a purely functional cDNA library screen for genes inhibiting apoptosis (Hashimoto et al., 2001). More recently, the MOTS-c peptide has been discovered in an sORF on the 12s rRNA gene in the mitochondrial genome by a bioinformatics screen of sORFs in mtDNA, showing a conserved 51nt ORF with a strong Kozak context (Lee et al., 2015). Treatment of HEK293 cells with a synthetic peptide substantially regulated gene expression of enzymes involved in cellular metabolism, and treatment of mice fed a high-fat diet prevented obesity, suggesting that the peptide is an extracellular signaling molecule.

## Viral Genome Annotation by Micropeptide Analysis

Finally, searches of viral genomes for micropeptides have also yielded some interesting results. Due to the small size of many of their genomes, historically, viral genomes were not usually annotated with explicit lower limits on the lengths of open reading frames, although the vaccinia virus, which has a large genome, had a lower limit of 65 codons for its original annotation (Goebel et al., 1990). Even without explicit minima during annotation, however, many viral micropeptides were simply overlooked because of systematic assumptions of how large genes should be, as genomes were annotated in an *ad hoc* fashion (Ratner et al., 1985). Micropeptides in viral genomes have been discovered in a similarly *ad hoc* manner, with several micropeptides found in influenza virus, human immunodeficiency virus, papillomavirus, poxviruses, and paramyxoviruses (DiMaio, 2014). These micropeptides are often dominated by a single alpha-helical transmembrane domain, which allows them to be inserted into lipid bilayers, in which context they can interact with and regulate host cell proteins (i.e., HIV-1 Vpu), form ion-selective pores (i.e., influenza M2), allow binding and entry into host cells (i.e., poxvirus O3L), or perform other functions crucial for the viral life cycle (DiMaio, 2014). A genome-wide screen of human cytomegalovirus by ribosome profiling and mass spectrometry found ∼484 novel ORFs shorter than 80 codons, including 245 shorter than 20 codons which were actively translated by the ribosome (Stern-Ginossar et al., 2012). Similarly, a recent study has applied ribosome profiling methods to the novel coronavirus SARS-CoV-2 and was able to find evidence of 23 novel proteins, beyond the 37 proteins already annotated for the virus (Finkel et al., 2020). These micropeptides must be validated by functional studies, but the high number identified here shows that there may be a substantial number of micropeptides to be found in all viruses.

## How Many Novel Micropeptides Remain to Be Discovered?

It has been widely shown by a variety of methods, therefore, that despite underrepresentation in genome annotations, sORFs encode functional micropeptides in nearly all genomes studied, thus suggesting that there may be many micropeptides produced both from the human genome and from human pathogens that may be of relevance to human health. However, how many micropeptides there may be in genomes is still controversial. Many types of genome-wide approaches have given a wide range for how many coding micropeptides there are in the human genome, from tens of thousands to a few dozen (Andrews and Rothnagel, 2014). These problems reflect the issues identifying sORFs which caused them to be overlooked. In particular, it is difficult to detect evolutionary conservation of these ORFs, because their small size disrupts the statistical assumptions of homology detection algorithms such as BLAST or PhyloCSF (Couso, 2015). Thus, genome-wide searches for conservation of sORFs often incorrectly show them to be unconserved. Secondly, very short peptides were thought not to have stable secondary structure, and thus, if biological function is assumed to be entirely dependent on the structure of the protein, short peptides could be assumed not to have a function (Ingolia et al., 2014; Wright and Dyson, 2015). This lack of secondary structure can also be a problem for genome-wide description of sORF-encoded micropeptides, for example by mass spectrometry, since peptides without stable conformations may be rapidly degraded when cells are lysed for extraction (Hackett et al., 1986).

If these are reasons for micropeptide numbers to be underestimated historically, questions of artifact in newer methods may cause the number of functional micropeptides to be overestimated. In particular, each genome-wide method for micropeptide discovery can be prone to false positives for various reasons. Central methods which have been used to discover micropeptides genome-wide are ribosome profiling, mass spectrometry, RNA sequencing, and direct searches for conservation. In ribosome profiling, polysomes are extracted from cells, then treated with RNase to destroy RNA which is unprotected by ribosomes (Ingolia et al., 2009). The ribosome footprints are then sequenced, and these are used to map the positions of the ribosomes. However, failure of RNase to digest a given section of a transcript may be due to factors besides ribosome content; in particular, RNA secondary structure may also block digestion, and RNA may also be underdigested due to sub-optimal reaction conditions. RNA-binding proteins may also block digestion. Furthermore, the mere presence of ribosomes in a given ORF does not necessarily demonstrate translation of that ORF. Although certain features of ribosome profiling datasets, such as codon periodicity, can be used to determine “genuine” translation, this question continues to be controversial and as yet there is no definitive metric by which ORFs can be determined to be translated (Guttman et al., 2013; Ingolia et al., 2014; Calviello et al., 2016).

In RNA sequencing, the length of the transcript is sequenced, and the high sensitivity of this method has allowed many novel transcripts to be discovered. In particular, RNA sequencing allows the study of a large set of RNAs called long non-coding RNAs (Sun et al., 2013; Ulitsky and Bartel, 2013; Hart and Goff, 2016). These RNAs were originally believed to be non-coding because they contain no long ORF, but subsequent conservation analyses of short ORFs have revealed many of these lncRNAs to indeed be micropeptide encoding (Pauli et al., 2014; Anderson et al., 2015). Thus, lncRNAs, which continue to be discovered by improved deep sequencing methods, may therefore be a large source of novel micropeptides. However, for the reasons mentioned above, most mainstream conservation analyses cannot be well applied to sORFs, and thus, screening for conserved sORFs in lncRNAs to search for micropeptides likely underestimates the total number of sORFs. In an attempt to address this problem, a novel conservation detection method was developed, and some 2,000 novel ORFs were found in the genome (Mackowiak et al., 2015). However, only a small percentage of these could be confirmed by mass spectrometry or ribosomal profiling data. Thus, the number of micropeptides produced by newly discovered lncRNAs continues to be an open question. For these kinds of reasons, RNA sequencing experiments have not been able to provide a clear picture of how many micropeptides there may be in genomes.

Mass spectrometry for discovery of micropeptides also seems to run afoul of the underestimation problem. Firstly, as mentioned above, mass spectrometry attempts to directly detect micropeptides, and if it is true that micropeptides are rapidly degraded by proteolytic enzymes after cell lysis, mass spectrometry may have problems with the speed of degradation of micropeptides. Secondly, lncRNAs are known to be very low abundance in any given tissue (Cabili et al., 2015). Furthermore, very short peptides, shorter than 5 amino acids will not be detected by mass spectrometry, and very long peptides are also difficult to detect, although the latter problem may be solved by performing a trypsin digestion. There may also be biochemical issues with particular micropeptide sequences; for example, if the peptide contains no basic amino acids, it becomes difficult to detect by mass spectrometry. Thus, detecting micropeptides translated from lncRNAs by mass spectrometry may have problems with sufficient sensitivity. Indeed, across the literature, one finds a mismatch between deep-sequencing experiments, such as ribosome profiling, and mass spectrometry, with deep-sequencing based results generally producing much higher numbers of micropeptides detected than mass spectrometry. There are two possible explanations for this; first, mass spectrometry may underestimate, or deep sequencing may overestimate, the number of micropeptides in the genome, or both; second, ribosomes may bind to many ORFs promiscuously, but these peptides are either not translated or quickly degraded and non-functional. The latter implies that there is no clear evidence that there is any substantial number of micropeptides in the genome, but the former implies that there are an undetermined number of micropeptides remaining in the genome to discover.

## Conclusion

Micropeptide research has been a growing field for the past several years, and advances in technology have made it possible to investigate the nature of this so-called hidden genome within the known genome. However, much work remains to be done, both in functionally characterizing the many micropeptides that have already been found through various methods, and in searching for micropeptides in new sample types. Technologies will also need to continue to be improved in order to find all micropeptides that exist. Recent progress in this field, however, raises the possibility of an entirely new understanding of genome function.

## Author Contributions

All authors listed have made a substantial, direct, and intellectual contribution to the work and approved it for publication.

## Conflict of Interest

The authors declare that the research was conducted in the absence of any commercial or financial relationships that could be construed as a potential conflict of interest.
